# Association of Preoperative Gut Microbiota Disturbances with Postoperative Infectious Complications in Major Gastrointestinal Cancer Surgery: A Large-Scale Exploratory Study

**DOI:** 10.1245/s10434-025-18298-2

**Published:** 2025-09-12

**Authors:** Takuya Sugimoto, Yukihiro Yokoyama, Yukiko Kado, Shuta Yamamoto, Takashi Mizuno, Junpei Yamaguchi, Shunsuke Onoe, Tomoki Ebata, Takashi Asahara

**Affiliations:** 1 Department of Surgery, Division of Surgical Oncology, Nagoya University Graduate School of Medicine, Nagoya, Japan; 2 Yakult Central Institute, Yakult Honsha Co., Ltd., Kunitachi, Tokyo Japan

**Keywords:** Gastrointestinal surgery, Gut microbiota, Intestinal environment, 16S rRNA gene amplicon sequencing, Fecal lactic acid

## Abstract

**Background:**

Gut microbiota and intestinal environment are crucial in minimizing the risk for postoperative infectious complications of major gastrointestinal cancer surgery. However, comprehensive studies across multiple cancer types with large samples are limited. This study aimed to identify preoperative factors associated with the development of infectious complications after such surgeries.

**Methods:**

This large-scale exploratory study examined 381 patients who underwent surgery for seven types of gastrointestinal cancer. Preoperative fecal samples were analyzed for fecal organic acid concentrations using high-performance liquid chromatography and gut microbiota composition 16S rRNA gene amplicon-sequencing. The relationships between specific gut bacteria, lactic acid concentrations, and postoperative infectious complications were analyzed.

**Results:**

Fecal lactic acid concentrations were significantly higher in patients who experienced postoperative infectious complications, indicating that lactic acid concentration is an independent risk factor. Greater preoperative abundances of *Akkermansia muciniphila* and *Lactococcus lactis* were associated with a lower incidence of postoperative infectious complications. Additionally, *A. muciniphila* abundance was positively correlated with preoperative blood neutrophil and lymphocyte counts.

**Conclusions:**

Fecal lactic acid concentrations, *A. muciniphila*, and *L. lactis *were identified as factors associated with the development or suppression of postoperative infectious complications. These findings suggest novel avenues for predicting and managing infection risks through preoperative assessments and microbiota modulation.

**Supplementary Information:**

The online version contains supplementary material available at 10.1245/s10434-025-18298-2.

Recent advancements in gastrointestinal (GI) cancer surgery have led to notable improvements in surgical outcomes. However, postoperative infectious complications remain a significant challenge, adversely impacting patient prognosis even after curative resection.^1–3^ These complications can diminish quality of life, increase health care costs, delay initiation of adjuvant therapy, and raise perioperative mortality rates.^1,2,4^ Consequently, reducing the incidence of postoperative infections remains a critical priority in perioperative management. Although common strategies, such as antibiotic administration and nutritional management with immunomodulatory supplements,^5–7^ are used, they are insufficient to fully prevent these complications. Thus, an urgent need exists for the development of novel preventive strategies.

Gut microbiota plays a crucial role in various physiologic functions, including infection protection and regulation of the intestinal immune system.^8,9^ Recent studies have linked gut microbiota diversity and composition to postoperative infectious complications in patients with colorectal, esophageal, and gastric cancers.^10–12^ Organic acids, particularly short-chain fatty acids produced by gut bacteria, are vital for maintaining the intestinal environment, enhancing gut barrier function, and stimulating the immune response, thus contributing to protection against infection.^13,14^ Our previous research suggests that preoperative fecal organic acid profiles influence the development of postoperative infectious complications in patients undergoing hepatectomy with extrahepatic bile duct resection and subtotal esophagectomy.^15,16^ These findings emphasize the potential of targeting the gut microbiota and intestinal environment to reduce postoperative infectious complications. However, existing studies are limited by small samples, narrow disease types, and a focus on specific surgical procedures, highlighting the need for large-scale studies encompassing a broader range of GI cancers.

We hypothesized that identifying factors within the gut microbiota that contribute to postoperative infectious complications could aid in the development of new strategies for improving postoperative outcomes and identifying potential biomarkers to classify high-risk patients. To test this hypothesis, we aimed to analyze the relationship between gut microbiota composition and preoperative fecal organic acid concentrations and their association with the incidence of postoperative infectious complications among patients undergoing major GI cancer surgeries. A primary objective of this study was to assess whether this concept could be generalized to a heterogeneous surgical population, including those undergoing upper GI, lower GI, and hepato-pancreato-biliary procedures.

## Methods

### Patients

This large-scale exploratory study included 381 patients with a diagnosis of esophageal, gastric, biliary, hepatocellular, metastatic liver, pancreatic, or colorectal cancers at Nagoya University Hospital and two other affiliated hospitals. Specifically, the study enrolled patients scheduled to undergo GI surgery, with no specified exclusion criteria. These patients were scheduled for radical resection surgery between January 2019 and February 2022.

This study was conducted in accordance with the principles of the Declaration of Helsinki. The study was approved by the Human Study Ethics Committee of Nagoya University Hospital (approval no. 2018-0259). Written informed consent was obtained from all the patients before participation. This report was prepared following the Strengthening the Reporting of Observational Studies in Epidemiology (STROBE) guidelines.

### Surgical Management

Mild bowel preparation was administered to patients undergoing upper GI and hepato-pancreato-biliary (HPB) surgeries, whereas a more intensive regimen was used for those undergoing lower GI surgery. The perioperative antibiotic regimen and its duration (up to 2 days postoperatively) were standardized across all cases. Perioperative wound management, including intraoperative wound protection and irrigation before skin closure, was uniformly applied throughout the study. Additionally, postoperative glycemic control was managed with support from diabetes specialists.

### Recording of Clinical Data and Postoperative Complications

The postoperative course of each patient was monitored daily by an independent research assistant, and postoperative infectious complications were recorded up to 28 days postoperatively. Clinical data on pre-, intra-, and postoperative factors were extracted from the medical records. Postoperative infectious complications included pneumonia, wound infection, bacteremia (identified using conventional culture methods), intra-abdominal abscess, enteritis, and cholangitis. Pneumonia was diagnosed based on either positive sputum cultures or radiologic evidence of consolidation with fever (>38 °C) or leucocytosis (>12,000 white blood cells/mm^3^). Wound infection was defined as spontaneous or surgically released purulent discharge with positive cultures. Intra-abdominal abscesses were defined as the presence of purulent discharge with positive cultures from surgically placed abdominal drains or fluid collections necessitating postoperative drainage. Cholangitis was diagnosed based on the presence of sustained fever requiring antibiotic treatment, increased levels of serum hepatobiliary enzymes, and positive bile cultures, in the absence of other identifiable sources of infection.

Blood test data (albumin, C-reactive protein [CRP], and platelet, neutrophil, and lymphocyte counts) collected 1 to 2 days preoperatively were extracted from medical records. The neutrophil-to-lymphocyte ratio (NLR), a prognostic indicator in patients with cancer, was calculated as described previously.^17^

### Fecal Sample Collection

Fecal samples were collected 1 to 2 days before surgery, before initiation of bowel preparation. Approximately 1 g of feces was placed directly into two tubes: the one contained 2 mL of RNAlater (Thermo Fisher Scientific, Waltham, MA, USA) and zirconia beads, whereas the other was empty.^18^ The RNAlater sample was processed at 25 °C for 10 min after stirring and then stored at 4 °C, whereas the other tube sample was stored at − 80 °C.^18^ Subsequently, the tubes were transported to the Yakult Central Institute under the respective storage temperature conditions for further analysis.

### 16S rRNA Gene Amplicon-Sequencing

The fecal samples were pre-treated for DNA extraction, as described previously,^19^ and DNA was extracted using the QIAmp DNA Stool Mini Kit (Qiagen GmbH, Hilden, Germany). The 16S rRNA gene region-sequencing was performed as described previously.^19^ Briefly, the V1–V2 region of the 16S rRNA gene was amplified using forward 27Fmod2 and reverse 338R primers. Sequencing was performed on a MiSeq platform (Illumina, San Diego, CA, USA) using the MiSeq Reagent Kit v2 (Illumina). The amplicon sequence reads were processed using QIIME2 (version 2022.2; https://qiime2.org).^20^ Quality control was performed by denoising the sequences using the DADA2 plug-in, and raw sequence data were processed into a table of exact amplicon sequence variants (ASVs).^21^ Taxonomic information was annotated using the SILVA database (version 138.1, https://www.arb-silva.de/) and BLAST (https://blast.ncbi.nlm.nih.gov/Blast.cgi). Alpha diversity indices were estimated for 15,000 randomly selected sequences using the q2-diversity plug-in. Beta diversity indices were estimated as the Aitchison index using the DEICODE plug-in.

### Fecal Organic Acid Concentrations and pH

Fecal organic acid concentrations were measured using a high-performance liquid chromatography system equipped with a 432 Conductivity Detector (Waters Co., Milford, MA, USA) and two Shodex RSPak KC-811 columns (Showa Denko, Tokyo, Japan), as described previously.^18^ The lower limits of detection were as follows: 0.075 μmol/g for succinic acid, 0.2 μmol/g for lactic acid, 0.05 μmol/g for formic acid, 0.4 μmol/g for acetic acid, 0.5 μmol/g for propionic acid, 0.5 μmol/g for isobutyric acid, 0.55 μmol/g for butyric acid, 0.8 μmol/g for isovaleric acid, and 0.65 μmol/g for valeric acid. Fecal pH was measured by directly inserting the electrode of an IQ150 pH meter (IQ Scientific Instruments, Inc., Carlsbad, CA, USA).^18^

### Statistical Analyses

Statistical analyses were performed using R statistical software (version 4.3.2; R Foundation for Statistical Computing, Vienna, Austria: https://www.r-project.org). Continuous data are expressed as medians (interquartile ranges). Fisher’s exact test was used to analyze categorical data, and the non-parametric Wilcoxon rank-sum test was used to compare continuous data between the two groups. For data in which fecal organic acid concentrations were below the detection limit, half of the detection limit value was substituted. Logistic regression analysis was performed using the “glm” function in the “stats” R package, and cutoff values were calculated from receiver operating characteristic analysis using the “roc” function from the “pROC” R package.

Differences in beta diversity between groups were analyzed using a pairwise permutational analysis of variance test (PERMANOVA) with the “adonis” function in the “vegan” R package. Differentially abundant species-level gut microbes between the groups were identified using the “DESeq2” R package.^22^ Species detected in more than 20 % of all the patients were selected for analysis. The relationship between gut microbiota and preoperative factors was analyzed using multiple regression analysis, with the relative abundance of each gut bacterium as the dependent variable and gender, age, body mass index (BMI), cancer type, neoadjuvant chemotherapy, duration of operation, and blood loss included as covariates. The relative abundance of the gut microbiota was log-transformed using the centered log-ratio transformation with the “transform” function in the “microbiome” R package. Then, multiple regression analysis was performed using the “glm” function in the “stats” R package. Statistical significance was set at a *p* value lower than 0.05 for all analyses.

## Results

### Patient Characteristics

This study enrolled 381 patients (255 males [67 %] and 126 females [33 %]), including 68 patients with esophageal cancer, 33 with gastric cancer, 34 with hepatocellular cancer, 52 with metastatic liver cancer, 70 with biliary cancer, 77 with pancreatic cancer, and 47 with colorectal cancer (Table 1). The median age of the patients was 71 years (Table 1). Among these patients, 113 (30 %) received proton pump inhibitors, 22 (5.8 %) received preoperative antibiotics treatment for some infectious complications, and 123 (32 %) received neoadjuvant chemotherapy (Table 1). Preoperative biliary stenting was performed in 83 patients (22 %), all of whom were undergoing HPB surgery.

**Table 1:** Baseline characteristics of patients

Characteristics	(*n* = 381) *n* (%)
Median age: years (IQR)	71 (63–76)
Sex ratio (M:F)	255:126
Median BMI: kg/m^2^ (IQR)	22.1 (20.0–24.1)
*Cancer type*	
Esophageal	68 (18)
Gastric	33 (8.7)
Hepatocellular	34 (8.9)
Metastatic liver	52 (14)
Biliary	70 (18)
Pancreatic	77 (20)
Colorectal	47 (12)
*Preoperative medication*	
PPI	113 (30)
H_2_-receptor antagonist	9 (2.3)
Laxative	55 (12)
Antibiotics	22 (5.8)
Lactic acid bacteria preparation	108 (28)
Neoadjuvant chemotherapy	123 (32)
Postoperative infectious complications^a^	94 (25)
Pneumonia	17 (4.5)
Sepsis	9 (2.4)
Wound infection	30 (7.9)
Intra-abdominal abscess	21 (5.5)
Enteritis	8 (2.1)
Cholangitis	11 (2.9)

IQR, Interquartile range; BMI, Body mass index; PPI, Proton pump inhibitor

^a^Some patients experienced multiple postoperative infectious complications.

Postoperative infectious complications occurred in 94 patients (25 %), including wound infections in 30 (7.9 %), intra-abdominal abscesses in 21 (5.5 %), pneumonia in 17 (4.5 %), cholangitis in 11 (2.9 %), sepsis in 9 (2.4 %), and enteritis in 8 (2.1 %) (Table 1). A bias in the distribution of postoperative infectious complications was observed across cancer types (Table 2), with the highest incidence observed in patients with biliary cancer (47.1 %).

**Table 2:** Breakdown of postoperative infectious complications by cancer type

Cancer type	Pneumonia *n* (%)	Sepsis *n* (%)	Wound infection *n* (%)	Intra-abdominal abscess *n* (%)	Enteritis *n* (%)	Cholangitis *n* (%)	Incidence of postoperative infectious complications *n* (%)
Esophageal (*n* = 68)	10 (14.7)	0	7 (10.3)	2 (2.9)	3 (4.4)	0	22 (32.4)
Gastric (*n* = 33)	1 (3.0)	0	2 (6.1)	2 (6.1)	0	0	5 (15.2)
Hepatocellular (*n* = 34)	1 (2.9)	1 (2.9)	1 (2.9)	0	0	0	3 (8.8)
Metastatic liver (*n* = 52)	2 (3.8)	1 (2.6)	1 (2.6)	3 (5.8)	0	0	7 (13.5)
Biliary (*n* = 70)	1 (1.4)	6 (8.6)	8 (9.3)	8 (9.3)	2 (2.9)	8 (9.3)	33 (47.1)
Pancreatic (*n* = 77)	1 (1.3)	1 (1.3)	3 (3.9)	6 (7.8)	2 (2.6)	3 (3.9)	16 (20.8)
Colorectal (*n* = 47)	1 (2.1)	0	7 (14.9)	0	1 (2.1)	0	9 (19.1)

### Comparison of Pre- and Intraoperative Factors by the Incidence of Postoperative Infectious Complications

The patients who experienced postoperative infectious complications were significantly older (*p* = 0.013) and had a significantly lower BMI (*p* = 0.028) than those who did not (Table 3). Additionally, the patients with postoperative infectious complications had significantly lower preoperative albumin levels (*p* = 0.007) and significantly higher CRP levels (*p* = 0.005) (Table 3). Regarding intraoperative factors, the patients who experienced postoperative infectious complications had significantly longer operative times (*p* < 0.001), greater blood loss (*p* < 0.001), and a higher frequency of blood transfusions (*p* < 0.009) than those without complications (Table 3).

**Table 3:** Comparison of pre- and intraoperative characteristics between patients who had postoperative infectious complications and those who did not

Characteristics	Postoperative infectious complications		*p* value^a^
	No onset (*n* = 287) *n* (%)	Onset (*n* = 94) *n* (%)	
*Preoperative characteristics*			
Median age: years (IQR)	70 (62–76)	72 (67–77)	0.013
Sex ratio (M:F)	186/101	69/25	0.124
Median BMI: kg/m^2^ (IQR)	22.3 (20.2–24.3)	21.7 (19.4–23.7)	0.028
*Preoperative medication*			
PPI	84 (29.3 %)	29 (30.7 %)	0.771
H_2_-receptor antagonist	7 (2.4 %)	2 (2.1 %)	> 0.99
Laxative	31 (14.1 %)	13 (16.0 %)	0.679
Antibiotics	18 (6.3 %)	4 (4.3 %)	0.467
Lactic acid bacteria preparation	75 (26.1 %)	33 (35.1 %)	0.094
Neoadjuvant chemotherapy	91 (31.7 %)	32 (34.0 %)	0.674
*Preoperative blood parameters*			
Median albumin: g/dL (IQR)	4.00 (3.70–4.20)	3.90 (3.50–4.10)	0.007
Median CRP: g/dL (IQR)	0.10 (0.04–0.32)	0.14 (0.06–0.61)	0.005
Median platelet: ×1000/μL (IQR)	221 (174–268)	212 (169–266)	0.748
Median neutrophil: ×1000/μL (IQR)	3.25 (2.51–4.10)	3.65 (2.70–4.42)	0.083
Median lymphocyte: ×1000/μL (IQR)	1.40 (1.11–1.74)	1.30 (1.00–1.70)	0.157
Median NLR (IQR)	2.29 (1.65–3.25)	2.78 (2.06–3.58)	0.011
*Median intraoperative characteristics*			
Median of operation duration: min (IQR)	346 (217–460)	466 (384–532)	< 0.001
Median blood loss: mL (IQR)	208 (68–613)	615 (120–1,106)	< 0.001
Median blood loss per weight: mL/kg (IQR)	3.48 (1.13–10.41)	11.69 (2.34–20.20)	< 0.001
Intraoperative blood transfusion	40 (13.9 %)	24 (25.5 %)	0.009

IQR, Interquartile range; BMI, Body mass index; PPI, Proton pump inhibitor; CRP, C-reactive protein; NLR, Neutrophil-to-lymphocyte ratio

^a^*p* values were estimated using Fisher’s exact test or Wilcoxon rank-sum test.

### Comparison of Preoperative Fecal Organic Acid Concentrations by the Incidence of Postoperative Infectious Complications

No significant differences were observed in the total fecal organic acid concentration or in the concentrations of major gut short-chain fatty acids, such as acetic acid, propionic acid, and butyric acid, between the patients who experienced postoperative infectious complications and those who did not (Table 4). However, the preoperative lactic acid concentration was significantly higher in the patients who experienced postoperative infectious complications than in those who did not (*p* = 0.002; Table 4; Fig. S1).

**Table 4:** Comparison of preoperative fecal organic acid levels between patients who had postoperative infectious complications and those who did not^a^

Characteristics	Postoperative infectious complications		*p* value^b^
	No onset (*n* = 287)	Onset (*n* = 94)	
Total organic acids^c^	75.6 (56.2–105.3)	76.1 (55.4–105.2)	0.964
Succinic acid	0.84 (0.35–1.79) (*n* = 32)	0.99 (0.41–2.09) (*n* = 9)	0.207
Lactic acid	0.36 (0.13–1.30) (*n* = 124)	0.92 (0.13–2.02) (*n* = 28)	0.002
Formic acid	0.68 (0.30–1.09) (*n* = 38)	0.78 (0.34–1.33) (*n* = 16)	0.127
Acetic acid	43.7 (30.0–59.0) (*n* = 0)	41.7 (30.1–57.6) (*n* = 0)	0.739
Propionic acid	15.6 (10.6–21.5) (*n* = 1)	14.1 (10.1–20.4) (*n* = 6)	0.306
Isobutyric acid	1.57 (1.02–2.21) (*n* = 36)	1.46 (0.85–2.22) (*n* = 17)	0.444
Butyric acid	7.42 (4.27–11.6) (*n* = 8)	7.65 (4.15–11.0) (*n* = 7)	0.611
Isovaleric acid	2.03 (1.08–2.98) (*n* = 51)	1.87 (0.51–3.08) (*n* = 24)	0.467
Valeric acid	1.23 (0.33–2.12) (*n* = 102)	1.01 (0.33–2.47) (*n* = 44)	0.515
pH^d^	6.93 (6.48–7.49)	6.75 (6.38–7.30)	0.073

IQR, Interquartile range

^a^For data points with concentrations below the detection limit, values were imputed as half the detection limit.

^b^*p* Values were estimated using the Wilcoxon rank-sum test.

^c^Organic acid concentrations are shown as medians (IQR) of μmol/g of feces; the number of participants with concentrations of the relevant organic acids below the detection limit also is reported.

^d^pH is shown as the median (IQR).

### Effect of Preoperative Factors on the Incidence of Postoperative Infectious Complications

In the univariate analysis, CRP levels (>0.055 mg/dL; odds ratio [OR] 2.19; 95 % confidence interval [CI] 1.21–4.0; *p* = 0.011), NLR (2.19; OR 1.91; 95 % CI 1.10–3.38; *p* = 0.023), and preoperative fecal lactic acid concentrations (>0.62 μmol/g; OR 3.16; 95 % CI 1.80–5.68; *p* < 0.001) were significantly associated with an increased risk of postoperative infectious complications (Table 5). Furthermore, in the multivariate analysis, preoperative fecal lactic acid concentration remained significantly associated with the risk of postoperative infectious complications (OR 3.04; 95 % CI 1.71–5.52; *p* < 0.001; Table 5).

**Table 5:** Uni- and multivariate logistic regression analyses of the onset of postoperative infectious complications^a^

	Univariate			Multivariable		
	OR	95 % CI	*p* value	OR	95 % CI	*p* value
Albumin (>3.95 mg/dL)	0.860	0.50–1.47	0.581			
CRP (>0.055 mg/dL)	2.18	1.19–4.01	0.012	1.86	1.00–3.56	0.055
NLR (>2.19)	1.92	1.10–3.36	0.022	1.73	0.93–3.28	0.044
Fecal lactic acid (>0.62 μmol/g)	3.18	1.79–6.63	< 0.001	3.04	1.71–5.53	< 0.001

OR, Odds ratio; CI, Confidence interval; CRP, C-reactive protein; NLR, Neutrophil-to-lymphocyte ratio

^a^Coefficients were estimated using age, sex, body mass index, cancer type, neoadjuvant chemotherapy, duration of operation, and blood loss as covariates. The optimal cutoff value was estimated using receiver operating characteristic curve analysis.

### Comparison of Preoperative Fecal Lactic Acid Concentrations by Cancer Type

Among the six cancer types, excluding pancreatic cancer, the patients who experienced postoperative infectious complications tended to have higher preoperative fecal lactic acid concentrations than those who did not (Table 6). Moreover, the patients with gastric and colorectal cancers who experienced postoperative infectious complications exhibited particularly elevated lactic acid concentrations (Table 6).

**Table 6:** Comparison of preoperative fecal lactic acid concentrations between patients who had postoperative infectious complications and those who did not by cancer type

Cancer type	Postoperative infectious complications				*p* value^a^
	No onset		Onset		
	*n*	Concentration Median μmol/g (IQR)	*n*	Concentration Median μmol/g (IQR)	
Esophageal	50	0.13 (0.13–0.63)	18	0.67 (0.13–1.50)	0.039
Gastric	28	1.45 (0.68–2.50)	5	2.58 (1.22–4.60)	0.314
Hepatocellular	30	0.13 (0.13–0.59)	4	1.50 (1.09–3.03)	0.082
Metastatic liver	46	0.13 (0.13–0.69)	6	0.88 (0.25–2.36)	0.112
Biliary	37	0.13 (0.13–1.69)	33	0.93 (0.13–2.03)	0.124
Pancreatic	57	0.48 (0.13–1.18)	20	0.34 (0.13–1.49)	0.870
Colorectal	39	1.09 (0.54–2.37)	8	1.67 (0.94–2.75)	0.314

IQR, interquartile range

^a^*p* Value were estimated using the Wilcoxon rank-sum test.

### Comparison of Gut Microbiota by the Incidence of Postoperative Infectious Complications

**Fig. 1:**
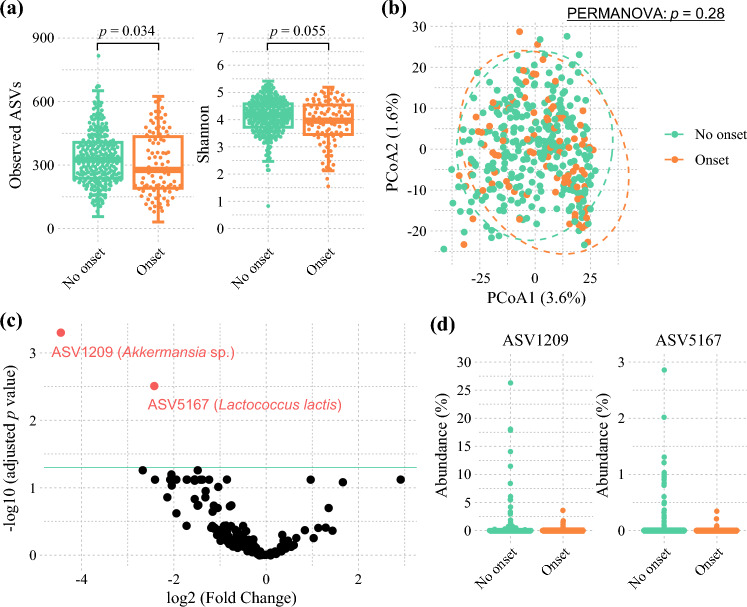
Comparison of the preoperative gut microbiota between patients who had postoperative infectious complications and those who did not. **a** Alpha diversity and **b** beta diversity based on the Aitchison distance compared between patients who had postoperative infectious complications and those who did not. For alpha diversity,* p* values were estimated using the Wilcoxon rank-sum test. For beta diversity,* p* values were estimated using the pairwise permutational analysis of variance (PERMANOVA) test. **c** Volcano plot of differentially abundant taxa between patients who had postoperative infectious complications and those who did not, assessed using DESeq2. Black points represent amplicon sequence variants (ASVs) with no significant difference in abundance, and red points represent ASVs with a significant difference in abundance (*p* < 0.05). **d** The abundance of significantly different taxa using DESeq2 compared between patients who had postoperative infectious complications and those who did not.

The patients who experienced postoperative infectious complications exhibited significantly lower observed ASVs (*p* = 0.034) and a trend toward a lower Shannon index, although the difference was not statistically significant (*p* = 0.055) compared with those who had no complications before surgery (Fig. 1a). However, no significant differences in beta diversity were observed between the two groups (Fig. 1b). Although slight but statistically significant differences in gut microbiota diversity were noted among different cancer types (Fig. S2), no significant differences were observed at the genus level (Fig. S3).

A comparison of the gut microbiota composition showed that the patients without postoperative infectious complications had a significantly higher abundance of ASV 1209 and ASV 5167 than those who experienced complications before surgery (Fig. 1c and d). Based on the SILVA database, ASV 1209 was identified as *Akkermansia* sp. and ASV 5167 as *Lactococcus lactis*. Furthermore, ASV 1209 was identified as *Akkermansia muciniphila* using a BLAST search.

### Association Between the Gut Microbiota and Preoperative Factors

The preoperative abundance of ASV 1209 (*Akkermansia* sp.) was significantly associated positively with the preoperative neutrophil and lymphocyte counts (Table 7). In contrast, no significant preoperative factors were associated with ASV 5167 (*L. lactis*).

**Table 7:** Association between the gut microbiota and preoperative characteristics

Characteristics	Partial regression coefficient (B)^a^		Standardized partial regression coefficient (β)^a^	*p* value
	Estimate (SE)	95 % CI		
*ASV 1209*				
Albumin	0.008 (0.009)	−0.010 to 0.026	0.041	0.386
CRP	0.016 (0.025)	−0.032 to 0.065	0.036	0.506
Platelet	3.738 (1.853)	0.105 to 7.372	0.044	0.044
Neutrophil	0.112 (0.032)	0.009 to 0.215	0.112	0.036
Lymphocyte	1.024 (0.272)	0.489 to 1.561	0.196	<0.001
NLR	0.026 (0.029)	−0.032 to 0.083	0.032	0.379
Lactic acid	−0.074 (0.068)	−0.207 to 0.059	−0.047	0.282
*ASV 5167*				
Albumin	−0.005 (0.010)	−0.024 to 0.051	−0.024	0.633
CRP	0.019 (0.027)	−0.034 to 0.071	0.037	0.482
Platelet	0.154 (2.03)	−3.815 to 4.124	0.004	0.939
Neutrophil	0.085 (0.035)	−0.011 to 0.124	0.092	0.102
Lymphocyte	−0.039 (0.304)	−0.634 to 0.555	−0.007	0.897
NLR	0.059 (0.032)	−3.210 to 0.121	0.097	0.064
Lactic acid	−0.077 (0.074)	−0.224 to 0.064	−0.054	0.302

SE, standard error; CI, confidence interval; ASV, amplicon sequence variant; CRP, C-reactive protein; NLR, neutrophil-to-lymphocyte ratio

^a^Coefficients were estimated using multiple linear regression analysis, with age, sex, body mass index, cancer type, neoadjuvant chemotherapy, duration of operation, and blood loss as covariates.

## Discussion

In this study, we performed a large-scale analysis involving 381 patients who underwent resection for any of the seven GI cancer types (esophageal, gastric, hepatocellular, metastatic liver, biliary, pancreatic, or colorectal) and evaluated whether our previous findings were broadly applicable across major GI cancer surgeries.^15,16^ Our results demonstrated a significant association between preoperative fecal lactic acid concentrations, the abundance of *A. muciniphila* and *L. lactis*, and the development of postoperative infectious complications.

The pKa of lactic acid is 3.86, lower than that of other organic acids.^23^ Consequently, lactic acid accumulation decreases intestinal pH, which alters the gut microbiota by inhibiting the growth of harmful gram-negative bacteria, such as *Escherichia coli*.^24^ However, excessive lactic acid accumulation in the intestine can have adverse effects.^25–27^ For example, in patients with short bowel syndrome, the inability of the shortened small intestine to absorb sugars enables gut bacteria to produce D-lactic acid, leading to life-threatening lactic acidosis.^27^ Elevated lactic acid levels have been observed in patients with severe ulcerative colitis.^26^ Furthermore, lactic acid serves as an energy source for pathogenic bacteria, such as *Salmonella enterica* Typhimurium, which cause intestinal infections.^25^ These findings suggest that lactic acid accumulation in patients with GI cancer may worsen intestinal conditions, promote systemic accumulation, and facilitate the growth of pathogenic bacteria, thereby increasing the risk of postoperative infectious complications.

Lactic acid in the gut is primarily produced by lactic acid-producing bacteria, such as *Lactobacilli* and *Bifidobacteria*.^28^ Under normal conditions, lactic acid is rapidly metabolized by obligate anaerobes, maintaining appropriate levels.^29^ However, patients with cancer often experience dysbiosis, disrupting gut microbiota composition.^30,31^ The lactic acid accumulation observed in this study was likely attributable to dysbiosis, potentially resulting from an overgrowth of lactic acid-producing bacteria or a reduction in lactic acid-utilizing bacteria.

In contrast, elevated lactic acid levels were observed in the patients with gastric and colorectal cancer despite only slight reductions in gut microbiota alpha diversity (Fig. S1). Additionally, there were no significant differences in the abundance of obligate anaerobes that utilize lactic acid or in the prevalence of lactic acid-producing bacteria, such as *Lacticaseibacillus*, *Lactobacillus*, *Ligilactobacillus*, *Limosilactobacillus*, *Enterococcus*, and *Streptococcus* (Fig. S2). The intestinal barrier function in patients undergoing perioperative cancer treatment is known to be compromised,^32,33^ and these patients are at increased risk of lactic acidosis due to factors such as ischemic tissue, reduced tissue perfusion, metabolic changes, bacteremia, and impaired liver function.^34^ These findings suggest that the elevated preoperative intestinal lactic acid levels observed in this study may result from two potential mechanisms: (1) increased production and reduced utilization by the gut microbiota and (2) translocation of accumulated lactic acid from the bloodstream into the intestinal tract. Further studies are needed to clarify the mechanisms underlying these changes in perioperative patients.

Dysbiosis in patients with cancer weakens the intestinal epithelial barrier and immune function.^35^ Moreover, it promotes the abnormal proliferation of pathogens and bacterial translocation, which subsequently induces postoperative infectious complications.^32,33,36,37^ Fecal lactic acid concentration reflects dysbiosis, the metabolic capacity of the gut microbiota, intestinal barrier integrity, and overall systemic condition. Thus, measuring the preoperative fecal lactic acid levels is more effective in predicting postoperative infectious complications than evaluating dysbiosis alone. Although blood parameters, such as the lymphocyte-monocyte ratio, CRP, and NLR, have been proposed as markers for predicting these complications,^38–40^ fecal lactic acid can be readily assessed using high-performance liquid chromatography with simple, non-invasive sampling, even by patients themselves. Therefore, this method could become a versatile tool for predicting infectious complications in clinical settings.

*Akkermansia muciniphila*, in particular, enhances intestinal epithelial barrier function.^41^ Maintaining this barrier has been linked to a reduced risk of postoperative infectious complications, likely due to its role in preventing bacterial translocation during GI surgery.^37,38,42^ It is plausible that a higher preoperative abundance of *A. muciniphila* strengthens the intestinal epithelial barrier, reducing the risk of postoperative infectious complications. Additionally, *Lactococcus lactis*, commonly found in dairy products,^43^ may have colonized the gut of patients who regularly consume such foods. In a respiratory infection mouse model, *Lactococcus lactis* administration enhanced intestinal barrier function and promoted transforming growth factor-β production, thereby reducing infection.^44^ Hence, increasing preoperative gut abundance of *L. lactis* may aid in reducing the risk of postoperative infectious complications in patients with GI cancer.

This study found no association between fecal lactic acid concentration and the abundance of *A. muciniphila* or *L. lactis*. It is unlikely that the abundance of these microbes directly affects lactic acid utilization and production in the intestine. The fluctuations in lactic acid levels cannot be solely attributed to individual gut microbes.

*A. muciniphila* plays a critical role in immune function by inducing adaptive immune responses in the gut, contributing to the maintenance of T cell homeostasis.^45^ In chemotherapy-induced enteritis models, *A. muciniphila* administration reduced the production of inflammatory cytokines, enhanced intestinal barrier function, and mitigated the reduction of neutrophils in the blood.^46^ These findings suggest that *A. muciniphila* enhances host resistance to pathogenic bacteria that cause infectious complications by modulating the immune function. Therefore, the prophylactic administration of *A. muciniphila* as a probiotic may reduce the risk for the development of postoperative infectious complications.

This study had some limitations. First, although it included patients with various cancer types, differences in preoperative management and chemotherapy varied, introducing potential confounding effects on microbiota. Nevertheless, the large scale of the study facilitated the identification of gut bacteria potentially involved in mitigating postoperative infectious complications.

Second, as an exploratory study, future validation of our findings is necessary, particularly regarding the sensitivity and specificity of fecal lactic acid in predicting postoperative infectious complications,. Additionally, the mechanisms whereby preoperative fecal lactic acid levels and the abundance of *A. muciniphila* and *L. lactis* contribute to postoperative infections remain to be fully elucidated. Moreover, effective strategies to maintain optimal preoperative fecal lactic acid levels have not been established to date. Therefore, further prospective studies are needed to clarify these mechanisms. Metagenomic analysis may be useful for elucidating the functionality of the aforementioned bacteria

Finally, because this study was conducted in a single population, similar analyses in other populations are necessary to confirm generalizability. Additionally, it is important to assess the applicability of these findings to surgical procedures beyond GI cancer resection and to consider the potential influence of other factors.

In conclusion, we identified preoperative fecal lactic acid concentration as a critical risk factor for postoperative infectious complications in major GI cancer surgeries. Furthermore, an increased abundance of *A. muciniphila* and *L. lactis* in the gut was associated with a lower incidence of these complications, suggesting a protective role for these microbes. These findings support using preoperative fecal lactic acid levels as a predictive marker and highlight the potential of administering *A. muciniphila* and *L. lactis* to mitigate infection risks. Further validation could lead to strategies to modulate the gut microbiota to reduce postoperative infectious complications.

## Supplementary Information

Below is the link to the electronic supplementary material.

Supplementary file1 (DOCX 483 KB)

## Data Availability

16S rRNA gene sequencing data in this study are available from the DDBJ Sequence Read Archive (DRA) under accession number DRA017242. The other data supporting the findings of this study are available from the corresponding author upon reasonable request.
