# Supplementary material for: Association of Preoperative Gut Microbiota Disturbances with Postoperative Infectious Complications in Major Gastrointestinal Cancer Surgery: A Large-Scale Exploratory Study
Source: Ann Surg Oncol. 2025 Sep 12;33(1):224–34. doi: 10.1245/s10434-025-18298-2 (PMC12689735; doi:10.1245/s10434-025-18298-2)
Supplement: Supplementary file 1 — Supplementary file1 (DOCX 483 KB) [file 10434_2025_18298_MOESM1_ESM.docx]

**Supplemental Online Figures**


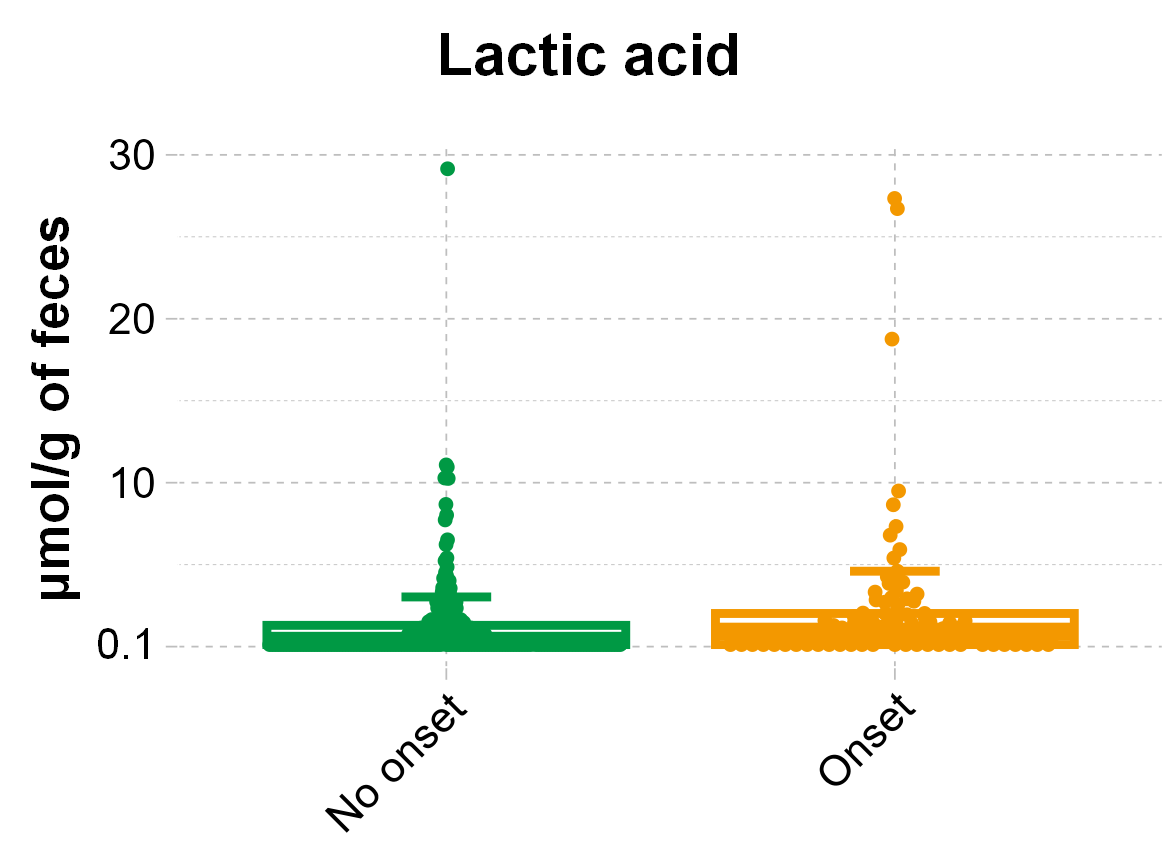


**Supplemental Figure S1.** Comparison of preoperative fecal lactic acid levels in patients with and without postoperative infectious complications.


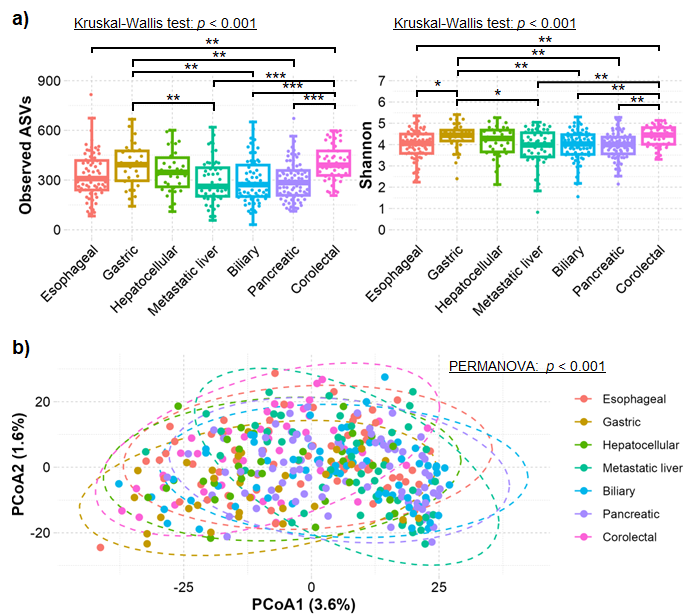


**Supplemental Figure S2.** Comparison of gut microbiota diversity among cancer types. (a) Alpha diversity and (b) beta diversity based on the Aitchison distance were compared among patients according to cancer type. For alpha diversity, *p*-values were estimated using the Kruskal–Wallis and post-hoc Dunn tests. For beta diversity, *p*-values were estimated using pairwise permutational analysis of variance (PERMANOVA).


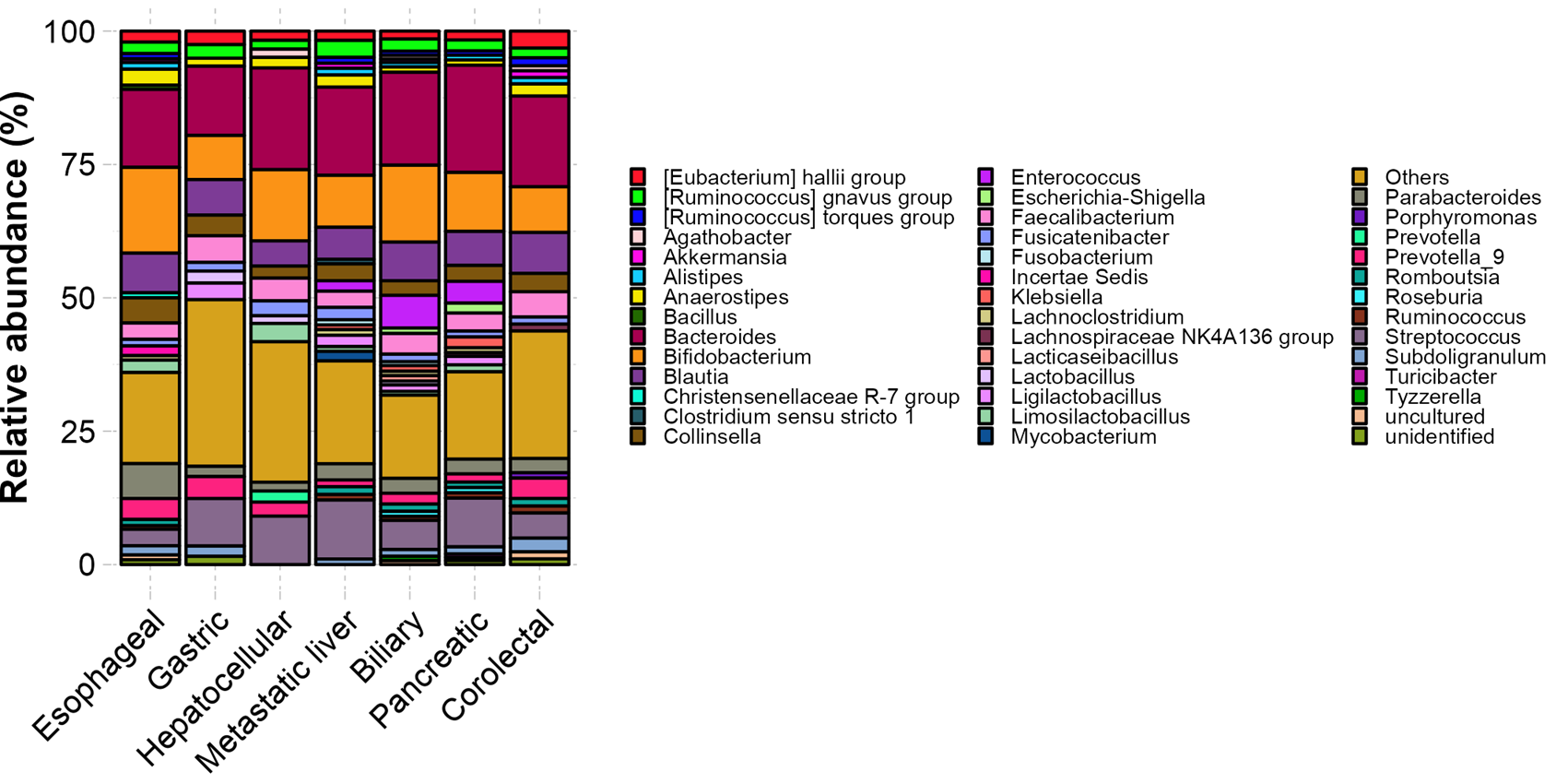


**Supplemental Figure S3.** Comparison of the gut microbiota among different cancer types. The abundance of the gut microbiota at the genus level was compared among patients according to the cancer type.
